# The Unintended Impact of Colombia’s Covid-19 Lockdown on Forest Fires

**DOI:** 10.1007/s10640-020-00501-5

**Published:** 2020-08-10

**Authors:** Mónica Amador-Jiménez, Naomi Millner, Charles Palmer, R. Toby Pennington, Lorenzo Sileci

**Affiliations:** 1 School of Geographical Sciences, University of Bristol, Bristol, UK; 2 Department of Geography and Environment, Latin America and Caribbean Centre and Grantham Research Institute on Climate Change and the Environment, London School of Economics (LSE), London, UK; 3 Department of Geography, University of Exeter, Exeter, UK; 4 Royal Botanic Garden, Edinburgh, UK; 5 Department of Geography and Environment and Grantham Research Institute on Climate Change and the Environment, London School of Economics (LSE), London, UK

**Keywords:** Armed groups, Covid-19, Colombia, Deforestation, Forest fires, Lockdown, Q23, Q56, Q58

## Abstract

The covid-19 pandemic led to rapid and large-scale government intervention in economies and societies. A common policy response to covid-19 outbreaks has been the lockdown or quarantine. Designed to slow the spread of the disease, lockdowns have unintended consequences for the environment. This article examines the impact of Colombia’s lockdown on forest fires, motivated by satellite data showing a particularly large upsurge of fires at around the time of lockdown implementation. We find that Colombia’s lockdown is associated with an increase in forest fires compared to three different counterfactuals, constructed to simulate the expected number of fires in the absence of the lockdown. To varying degrees across Colombia’s regions, the presence of armed groups is correlated with this fire upsurge. Mechanisms through which the lockdown might influence fire rates are discussed, including the mobilisation of armed groups and the reduction in the monitoring capacity of state and conservation organisations during the covid-19 outbreak. Given the fast-developing situation in Colombia, we conclude with some ideas for further research.

## Introduction

The global spread of covid-19 in 2020 has had, and continues to have, a devastating impact on our societies and economies. In response, governments have intervened on a huge scale to try to slow and manage the spread of the disease, help those who get infected, and support economies. With the aim of slowing the spread of disease, mandatory ‘shelter-in-place’ restrictions on peoples’ movements, also known as lockdowns or quarantines, typically prevent people from leaving their homes or local areas for extended periods of time. By April 2020, lockdowns had become one of the commonest policy responses to covid-19, affecting up to two-thirds of the global population (Bates et al. 2020).

Evidence is emerging that suggests lockdowns have unintended environmental consequences, both negative and positive. For example, research undertaken in China suggests that lockdowns are associated with improvements in local air quality, likely due to sharp falls in road traffic and manufacturing activity (e.g. Liu et al. 2020; Le et al. 2020), while a lockdown-induced collapse in ecotourism revenues may have negatively affected local livelihoods, leading to an increase in wildlife poaching (The Guardian 2020). Yet, the evidence base and hence, our understanding of how lockdowns might influence natural resource use, management and conservation, is still relatively weak. Our paper is an early, exploratory contribution, motivated by the release of satellite data showing a particularly large upsurge of forest fires in the Colombian Amazon that coincided with the emergence of covid-19 in Latin America, in early-2020 (IDEAM 2020; FCDS 2020a).

We ask whether Colombia’s lockdown, which was implemented in stages between 14 March and 24 March 2020 and is projected to end on 15 July (at the time of writing in early-July), is associated with this observed upsurge of forest fires. As detailed in Sect. 2, forest governance and conservation in Colombia are intimately associated with the country’s long history of internal conflict and the militarisation of conflict areas. Thus, any analysis of forest change in Colombia needs to consider the role of its numerous armed groups. This we do by first exploiting spatial variation in the known locations of armed groups across Colombia in our econometric analysis, the methods for which are described in Sect. 3.

The observed number of forest fires are compared with three different counterfactuals (historical average, synthetic control, augmented synthetic control), constructed to simulate the expected number of fires in the absence of Colombia’s lockdown. Our results, presented in Sect. 4, suggest that the lockdown is associated with an increase in the number of fires. To varying degrees across Colombia’s regions, the presence of armed groups is found to be correlated with this increase. How and why Colombia’s lockdown might influence forest fires are questions that cannot be addressed by our econometric analysis. Therefore, in Sect. 5, we consider a number of possible mechanisms, including the mobilisation of Colombia’s armed groups and the monitoring capacity of state and conservation organisations, based on information from local stakeholders in lowland and Andean Colombia and key informants connected with the Colombian Amazon, before and during the lockdown. The final section of the paper concludes with some ideas for further work.

## Background to Deforestation, Conflict and Covid-19 in Colombia

Colombia is one of the most biodiverse countries in the world, with five major biotic regions: Amazon, Andes, Caribbean, Orinoco and Pacific (Appendix Fig. 8). Between 1990 to 2016, more than six million hectares of natural forests were deforested (IDEAM 2018). There was a surge in deforestation after a peace agreement with the guerrilla movement FARC-EP (The Revolutionary Armed Forces of Colombia—People’s Army) was signed in 2016 (e.g. Clerici et al. 2020). The joint efforts of environmental organizations, state authorities, international cooperation, the media and communities helped reduce the annual deforestation rate nationally, by 10.1% in 2018, a trend that continued into 2019 (IDEAM 2018, 2020).

In common with many other parts of tropical Latin America, trees in Colombia are felled before any remaining forest is cleared by fire in preparation for new areas of crop cultivation and cattle pasture, although logging is not always followed by forest clearance via fire. This procedure takes advantage of seasonal climates, with felling often occurring in the wet season and fires subsequently started after weeks or months of less rain in the dry season. Most forest fires in the Colombian Amazon take place in the dry season, between November and April. When these dry season fires are started they can spread in forest areas where logging has not taken place previously. Forest fires are typically started by farmers or landless people seeking land for crop cultivation in order to feed their families and generate income, although other actors, such as armed groups, have also been implicated in forest fires.

Early estimates for 2020 suggest that rates of tree felling and deforestation in the Colombian Amazon are likely to reverse the gains of 2018 and 2019 (MAAP 2020; SINCHI 2020), while Colombia’s forest fire trends in the first half of 2020 imply that the country is on course to record one of its largest numbers of forest fires in recent years. As detailed in Sect. 3, a huge increase in the number of forest fires was observed in March 2020 (12,953) compared to March 2019 (4691) (Semana Sostenible 2020). One explanation is that more rain than usual fell in December 2019, with Colombia’s dry season starting later, in mid-January 2020 (FCDS 2020a).

Forest governance and conservation in Colombia are associated with the militarisation of conflict areas. The current internal armed conflict dates back to a period called ‘the Violence’, which lasted from 1948 until 1958 (Guzmán Campos et al. 2005). After this period, bitterness at the lack of attention from the government in dealing with the conflict’s underlying causes (land distribution and political exclusion), led to the rearming of guerrillas associated with the Liberal party and the conversion of some of these guerrillas into FARC-EP. Paramilitary groups were legally formed under the government’s auspices in opposition to the guerrillas. The emergence of the paramilitary groups was closely connected to the geographies of drug trafficking in Colombia (e.g. Cubides 1997; Gallego 1990; Vargas 1992). Areas under guerrilla control were branded ‘red zones’ while the areas controlled by the paramilitaries were seen as zones of special control and military presence. People living in these areas came under the rule of these armed groups and often endured terrible hardship.

After the peace agreement with FARC-EP in 2016, the ELN (National Liberation Army) guerrillas, along with dissidents from FARC-EP, heavily-armed organized criminal organizations and neo-paramilitary groups associated with the political far right (see below),^1^ expanded their territorial control in several forest areas, taking over areas that were previously controlled by FARC-EP. In some parts of Colombia, individual armed groups have secured full territorial control while in other parts different groups have been disputing control.

Until 2018, there was a rise in coca cultivation and deforestation rates in the Amazon, attributed to a lack of active government presence after the peace process (DeJusticia 2018). The inability of the government in the post-conflict era to fill the power vacuum in areas previously occupied by FARC-EP, gave rise to the emergence of neo-paramilitary groups: the Urabeños, the Rastrojos and the Gulf Clan. Implicated in drug trafficking in association (or dispute) with Mexican drug cartels (PARES 2018; FIP 2019), these groups tended to operate on the Pacific coast and in some Inter-Andean areas.

Many areas contested by the armed groups were also priority areas for the government’s military and conservation strategies. After 2016, the Colombian government promoted sustainable development and ecotourism, in an attempt to transform areas that were heavily affected by the armed conflict and previously under the full or partial control of FARC-EP. Under these government- and NGO-led schemes, some of which were implemented in National Natural Parks and areas of high biodiversity, former guerrillas were encouraged and trained for new roles, e.g. as forest guardians, tourist guides and organic farmers.^2^

The reduction in deforestation rates in 2018 and 2019 is partially attributed to Operation Artemisa, an initiative to curb deforestation in protected areas, led by the armed forces with the support of the Chief Prosecutor’s Office, the Ministry of the Environment, and the Institute for Hydrology, Meteorology and Environmental Studies. Using satellite data, Geographical Information Systems, drones and field intelligence, some operations led to the arrest of actors, often local farmers and the landless, caught deforesting and starting fires illegally. Yet, the militarisation of the environmental agenda led to new rounds of conflict and protest. Peasant organizations, environmental NGOs and human rights organizations have alleged that excessive force was used against local farmers and the landless, who were often treated as guerrilla collaborators. None of those financing and organizing the deforestation and forest fires, namely members of the armed groups, have been detained.

Colombia’s first case of covid-19 was reported on 6 March 2020. The government’s response to the outbreak that ensued was to implement a number of lockdown measures with the following timeline (Presidential Decree 749, 2020):

- 14 March: closure of border with Venezuela
- 15 March: suspension of all schools and universities
- 16 March: closure of all land and sea borders; curfews in several municipalities
- 17 March: declaration of state of emergency; mandatory isolation for all over-70s
- 20 March: announcement of nationwide quarantine, starting at midnight on 24 March

At the time of writing, Colombia’s quarantine for all of the country’s citizens was extended until 15 July, one the world’s most prolonged. Colombia’s lockdown was effective in reducing mobility at its onset, soon after the closing of its land border with Venezuela (see Appendix Figs. 9, 10). As of 7 July 2020, Colombia reported 124,494 cases of covid-19, of whom 4359 have died.

## Data and Methods

### Data Description

Our econometric analysis in Sect. 4 primarily utilises data for forest fires detected by the Visible Infrared Imaging Radiometer Suite (VIIRS) on board the Suomi NPP satellite. The spatial resolution of VIIRS is 375 metres resolution per pixel, a higher resolution than that of the Moderate Resolution Imaging Spectroradiometer (MODIS) sensor (1000 m resolution per pixel). Thus, VIIRS can detect fires that MODIS might overlook, although fire data from the latter are used as an additional predictor in our application of the synthetic control method (SCM; see below). Daily data for forest fires detected by both VIIRS and MODIS are sourced from the National Aeronautics and Space Administration (NASA).^3^

While MODIS data are available from 2001 onwards, VIIRS began operating in 2012; therefore, our data cover all of Colombia over the period between 1 January 2012 and 28 May 2020. We use the daily count of VIIRS and MODIS fire hotspots, a flow variable, and the daily cumulative sum of fire hotspots, a stock variable. Additional analysis makes use of the sum of daily Fire Radiative Power (FRP), also reported in the VIIRS and MODIS products, which accounts for heterogeneity in fire hotspots’ size and intensity. Fire hotspots are aggregated at the country level (for the analyses that cover all of Colombia), and the municipality level (allowing us to examine regional heterogeneity). Given heterogeneity in the biophysical and ecological characteristics of the country, including climatic conditions and forest types, the municipalities are grouped by biotic region.

**Fig. 1:**
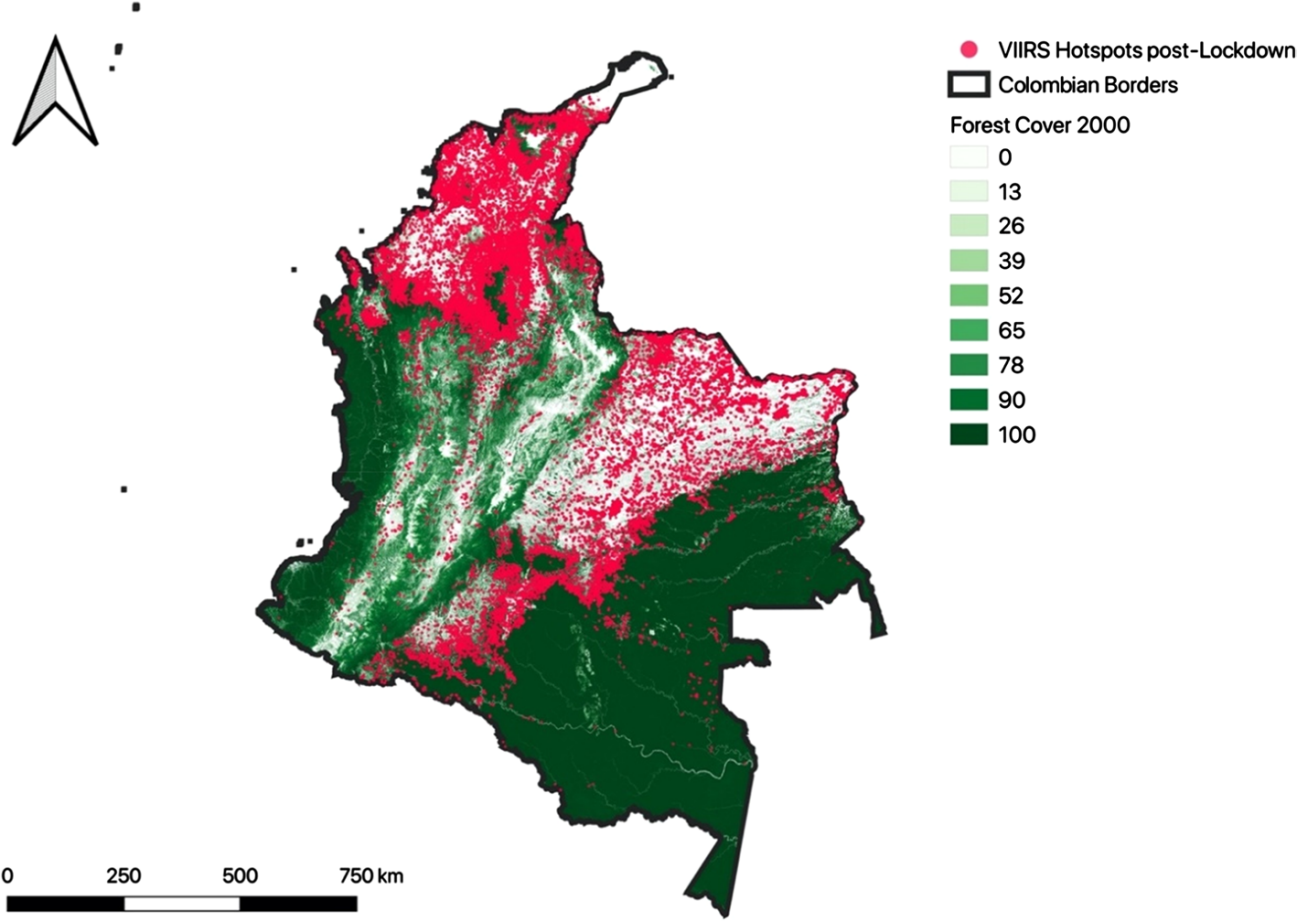
Location of fire hotspots in Colombia, 14 March–28 May 2020. *Sources*: Hansen et al. (2015), NASA Goddard Space Flight Center, Ocean Ecology Laboratory, Ocean Biology Processing Group, 2020. Visible and Infrared Imager/Radiometer Suite (VIIRS)

Figure 1 shows the location of fire hotspots during Colombia’s lockdown, up until 28 May 2020. Although fires can be observed across the country, in all biotic regions, they are particularly concentrated in northern, central and south-western areas. The northern and central zones are more seasonally dry, with large areas of dry forest and savanna that are naturally more fire-prone, but there are also many fires in the Andean valleys, and in and near the Amazon frontier. The lockdown restrictions mandated that the entire population of Colombia should stay home yet the patterns of fire hotspots in Fig. 1 suggest that people, at least in some parts of the country, were ignoring these restrictions.

**Fig. 2:**
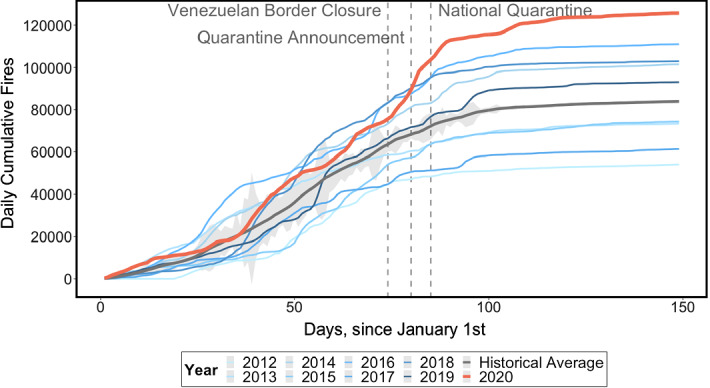
Cumulative number of forest fires by year in Colombia, 2012–2020. *Note*: 95% Confidence interval around the historical mean shaded in grey. *Source*: NASA Goddard Space Flight Center, Ocean Ecology Laboratory, Ocean Biology Processing Group, 2020. Visible and Infrared Imager/Radiometer Suite (VIIRS)

Our 2012 to 2020 time-period, while motivated by data limitations, implies a reasonable level of confidence in ruling out major climatic shocks as the sole drivers of extreme fire seasons. We expect to observe some yearly fire variability in our sample, although as shown in Figs. 2 (daily count of fires) and 3 (cumulative daily number of fires), the dispersion of the time series is relatively contained. There is, however, an unusual spike in fire hotspots in both the count and cumulative trends starting from 14 March 2020, when the border with Venezuela was closed, the first step in Colombia’s lockdown response to the covid-19 pandemic.

**Fig. 3:**
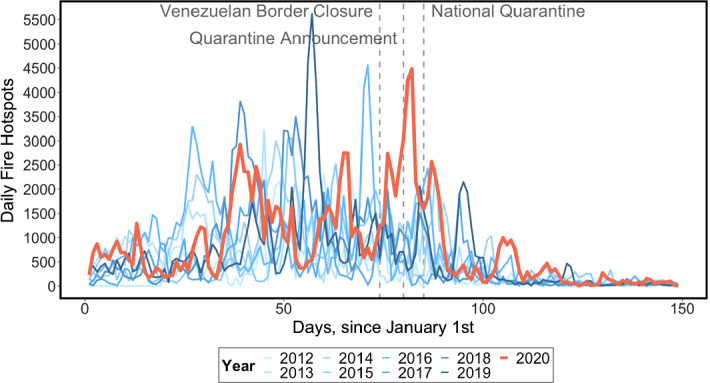
Daily number of forest fires by year in Colombia, 2012–2020. *Source:* NASA Goddard Space Flight Center, Ocean Ecology Laboratory, Ocean Biology Processing Group, 2020. Visible and Infrared Imager/Radiometer Suite (VIIRS)

We are unable to augment the dataset with the inclusion of relevant climatic covariates (temperature, rainfall, wind speed, etc.) as controls due to the near-real time nature of our analysis. Thus, a major caveat of our analysis is the possibility that any effects we find are mediated by extreme climatic shocks during the lockdown period or by an idiosyncratic alteration in the timing of the start of the dry season. Visual inspection of the trends in cumulative hotspots (Fig. 2) and fire counts (Fig. 3) does not lend full support to these alternative explanations. Indeed, the patterns for the 2020 fire season indicate spikes similar to earlier years prior to mid-March and an unusual upsurge in hotspots during the covid-19 lockdown. Also, 2020 was already the third-highest fire season in the record as of 14 March 2020, having deviated from the long-run historical mean since early-February, thereby reducing the likelihood that the observed increase in March was solely due to a late dry season.

Our discussion of how and why Colombia’s covid-19 lockdown might influence deforestation, presented in Sect. 5, is based on secondary sources, interview data and information obtained from research networks and key informants associated with the interdisciplinary research project ‘BioResilience: Biodiversity resilience and ecosystem services in post-conflict socio-ecological systems in Colombia’.^4^ Since 2018, extensive socio-cultural fieldwork has been conducted in two areas representative of two different socio-ecological systems in the central-eastern Andean mountain range: the lowlands of the Middle Magdalena region (lowland rain forest merging with lower montane (cloud) forest at higher altitudes, above 1500 m) and the highlands of the National Natural Park Chingaza.

### Methodology

We first estimate ‘excess fires’ over the lockdown period in Colombia covered in this paper, that is, from 14 March 2020 until 28 May 2020. Our measure of excess fires follows the methodology used to estimate excess mortality, a tool used in epidemiology to describe the number of deaths exceeding what would have been expected under ‘normal’ conditions. Specifically, we use the methodology used to calculate excess deaths due to covid-19 by The Economist and the Financial Times (for an overview, see Roser et al. 2020), and adapt it to our purposes. Thus, the number of excess forest fires, *EF*, during the lockdown period is calculated by subtracting the mean number of fires, *MF*, during this same period between 2012 and 2019, i.e. 14 March–28 May, from the total number of fires observed, *OF*, between 14 March and 28 May, 2020:

$$EF_{{14\,{\text{March}} - 28\,{\text{May}},2020}} = OF_{{14\,{\text{March}} - 28\,{\text{May}},2020}} {-}MF_{{14\,{\text{March}} - 28\,{\text{May}},2012 - 2019}}$$

Therefore, our first empirical approach simply compares the observed number of fires against a historical average number of fires. As shown in Figs. 2 and 3, however, 2020 deviated from the mean historical fire trend well before the beginning of the lockdown, biasing this comparison upwards: cumulative fires on 14 March 2020 were already in excess of the 95% confidence interval around the long-run mean. Since this discrepancy could have been driven by idiosyncratic climatic factors specific to 2020, such a comparison is only useful for descriptive purposes, identifying this year’s fire season as anomalous with respect to prior ones. Thus, the historical mean of fire trends is not a statistically-grounded counterfactual for 2020 fire observations because it violates the foundational assumption of parallel trends prior to treatment.

To account for this problem, we apply two further approaches, akin to difference-in-differences, which compare the observed number of fires with a counterfactual constructed to simulate the expected number of fires in the absence of Colombia’s covid-19 lockdown. Both methods have advantages over the use of the historical average in terms of how the underlying distribution of the historical fire data is treated and in accounting for pre-lockdown time trends in forest fires. Moreover, both methods are geared towards the construction of a counterfactual that closely tracks fire trends in 2020, thereby ensuring that fire trends for the treated and control units are optimally matched for the whole pre-treatment period, conditional on the feasibility of said matching.

Our second approach is the SCM. First developed by Abadie and Gardeazabal (2003), the SCM estimates an artificial counterfactual for the single treated unit via a data-driven method that employs minimal assumptions on its underlying data distribution. A synthetic control, our counterfactual, is a weighted average of the available control units. The weights for these units are estimated by minimizing the difference between the counterfactual and what was actually observed during the pre-treatment period.

Following Modi et al. (2020), we construct the synthetic control unit for Colombia’s 2020 fire trends from a weighted average of fire trends in prior years. On the one hand, by using historical time periods in a single country rather than multiple countries, this procedure has the advantage of ruling out cross-country differences that cannot be summarised by the predictors of choice (e.g. different structures of the forestry sectors, differences in monitoring and enforcement, different timings of the wet and dry seasons). On the other hand, it is possible that events specific to 2020, perhaps not previously observed in the fire record, are entirely responsible for the deviation of the actual trend from its synthetic counterfactual.

Another concern arises from the inspection of the time series used as outcome variables. Indeed, as reported by Masini and Medeiros (2019), the SCM suffers from issues of over-rejection of the null hypothesis of no effect when the data are nonstationary, as is the case for the daily count of fire observations (Fig. 3). For this reason, we use the cumulative count of fire observations, rather than daily fire counts, as our outcome variable.

To partially account for the issues connected to the data-generating process encountered with the SCM, we adopt the augmented SCM (ASCM, Ben-Michael et al. 2019). Our third approach addresses a concern about the SCM, namely that it may not provide a meaningful estimate of excess forest fires if the trajectory of fires in the synthetic control unit does not closely match the trajectory of the lockdown treatment unit prior to the intervention (Abadie et al. 2015). In particular, we follow Ben-Michael et al. (2019) and Cole et al. (2020) in implementing a ridge-regularised outcome regression model to estimate and correct for the bias arising from discrepancies in pre-intervention fit between the treated and synthetic units. Also, the ASCM is able to describe dispersion around its point estimate by employing the ‘average squared placebo gap’, which makes use of the standard leave-one-out SCM estimates in calculating the SCM noise variance (Ben-Michael et al. 2019).

The synthetic and augmented synthetic controls are constructed by adapting the methodology from Modi et al. (2020) and employ 2012–2019 data as the ‘donor pool’. The number of pre-treatment days is $T = 73$. We employ MODIS fire observations, VIIRS Fire Radiative Power (FRP) and four lags of the dependent variable ($n = 6$) as predictors. Both approaches transparently report the observations receiving non-zero weights in the construction of the artificial counterfactuals. For the SCM, the 2020 trends are reconstructed from a weighted combination of trends recorded in 2018, 2013 and 2016.^5^ The ASCM algorithm allows negative weights to be placed on donor observations thus making them slightly less interpretable. Nonetheless, 2018 and 2013 are again the most important fire seasons used in the construction of the counterfactual, followed by 2019 and 2016.^6^

We perform two robustness checks on our country-level results. First, an in-time placebo test (Abadie et al. 2010, 2011, 2015) for both the SCM and ASCM, to check whether the optimisation algorithm provides significant results in the absence of an intervention. If a test of no intervention effects fails to reject the null hypothesis, then the method is poorly identified. We impose a placebo lockdown on 19 February that ends on 13 March, letting the matching procedure run up until 19 February. Second, we replicate the analysis using Fire Radiative Power (FRP) as an alternative dependent variable. By measuring the radiative intensity of fire hotspots, this outcome variable minimises the possibility that our results stem from more frequent, but less intense, fire observations.

To test for the number of forest fires conditional on the presence of armed groups in our regional analysis, data on the known locations of armed groups in Colombia are digitally coded into our dataset from maps originally created in 2019 by the Peace and Reconciliation Foundation (PARES). We focus on two of the main groups, with broad geographic reach in Colombia: FARC-EP dissidents and the Gulf Clan neo-paramilitaries (Appendix Fig. 11).

Qualitative insights presented in Sect. 5 were generated from fieldwork involving ethnographies, participant observation, interviews and workshops. Dialogue with the inhabitants of the highlands as well as with those of the lowlands continued during Colombia’s lockdown, via mobile phone and other electronic means. This dialogue, though not initially motivated by our research question, gives clear local perspectives on land-use change during the lockdown period, enabled by a high level of trust that has been built between local people, including community leaders, and members of the BioResilience research team. The BioResilience team is also embedded in research and civil society networks across the country, which have generated insights in other regions beyond the Andes, in particular, the Amazon. For security reasons, key informants and stakeholders are not cited in the text unless their views have already been made public, e.g. via NGO reports.

## Results

We provide exploratory, quantitative evidence for the unintended impact of Colombia’s lockdown on forest fires, beginning with our results for all of Colombia. The actual time series of cumulative fire observations in 2020 is examined vis-à-vis the 2012–2019 historical mean, the synthetic control counterfactual and an augmented synthetic control estimated via a ridge regression in the pre-treatment period.

**Fig. 4:**
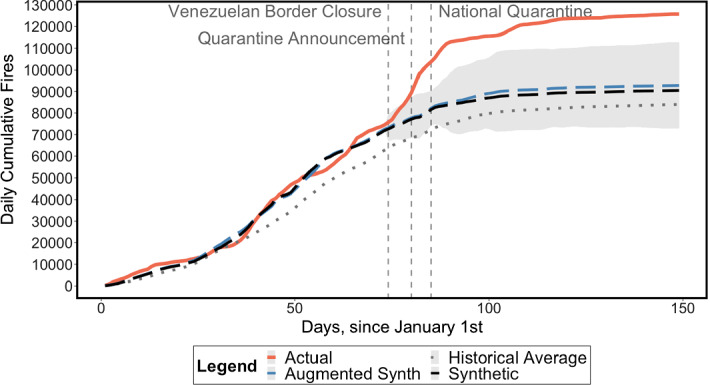
Actual series, historical mean, SCM and ASCM, for all of Colombia. *Note:* 95% Confidence Interval around the ASCM series shaded in grey. (Color figure online)

As noted in Sect. 3, the historical mean does not represent an adequate counterfactual for 2020 fire trends. Indeed, even if the 2020 series is located in the 95% confidence interval around the mean up until February (see Fig. 2), the trends diverge from January onwards, and exhibit fundamentally different slopes at the start of the treatment in mid-March. Thus, any comparison that uses the historical mean as a counterfactual substantially overestimates the upsurge in 2020 fires, as evidenced in Fig. 4, which shows the results of our country-level analysis.

The historical average is shown by the dotted line in Fig. 4 while the counterfactual fire trends generated by the SCM and ASCM are shown by the black and blue dashed lines, respectively. The lines generated by the SCM and ASCM clearly improve upon the simple historical mean. Indeed, these two fire trends are much more closely matched to actual fire observations (line shaded red) in 2020, up until 14 March, than the historical average. After 14 March, they diverge dramatically thus indicating evidence of a clear upsurge in cumulative daily fires. Note that the 95% confidence interval around the augmented synthetic control, shaded grey in Fig. 4, does not overlap with the 2020 actual series, thus identifying a statistically significant divergence of the 2020 fire season from weighted combinations of previous years’ fire rates.

As of 28 May, the discrepancy between the actual 2020 fire season and its synthetic counterfactual totals 35,212 fires, a number that falls to 13,019–52,781 (the point estimate is 32,900) with respect to the augmented synthetic control. Notably, 80.4% of this difference (28,396 fires) is recorded within 1 month from the closure of the border with Venezuela (58.6–84.6% or 7631–44,661 fires when employing the ASCM, with a point estimate of 78.7% or 25,894 fires), which indicates either that the lockdown created particular incentives to start forest fires and/or that specific climatic conditions have postponed the fire season to coincide precisely with the period of lockdown.

**Fig. 5:**
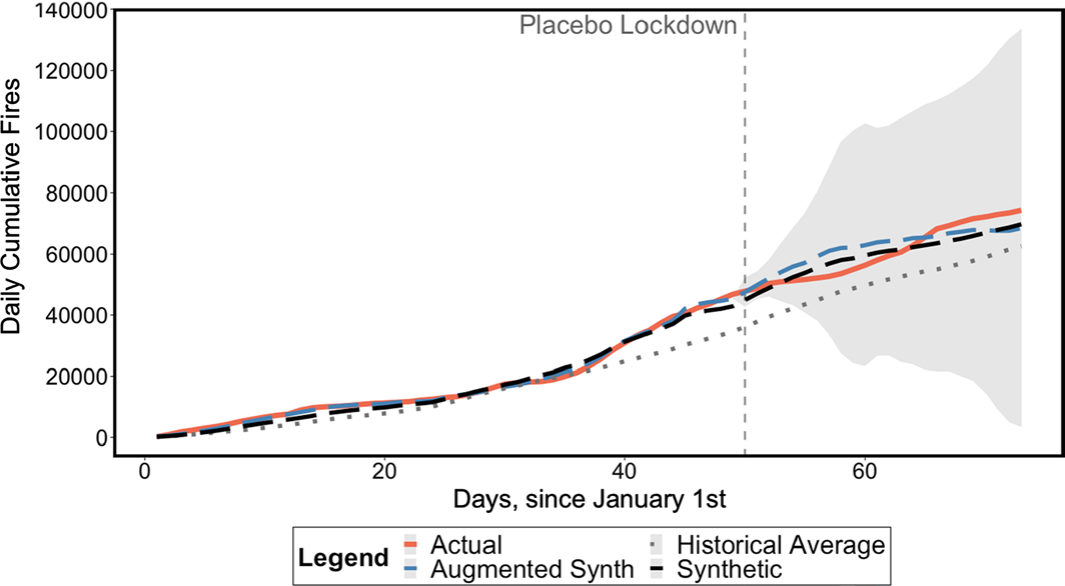
In-time Placebo Test for SCM and ASCM, with lockdown beginning on 19 February 2020. *Note*: 95% Confidence Interval around the ASCM series shaded in grey

Our two robustness checks provide support for our country-level results. First, the in-time placebo test results, shown in Fig. 5, suggest that both the SCM and ASCM fail to identify an upsurge in fires coinciding with the placebo lockdown period, between 19 February and 13 March, thereby validating our SCM and ASCM procedures. Second, results from replicating our analysis using Fire Radiative Power (FRP) as an alternative dependent variable (Appendix Fig. 12) are consistent with those in Fig. 4.

Figure 5 suggests that we are almost certainly capturing the pre-lockdown trend correctly. Yet, we remain somewhat cautious about our results in Fig. 4 due to the non-stationarity caveats raised by Masini and Medeiros (2019) and the unavailability of climatic data. Thus, we cannot completely discount the possibility that our results, while robust and significant, could be plagued by oversized tests of no intervention effects or, unlikely as it may seem, mediated by extreme climatic events coinciding with the lockdown.

Although the cumulative number of fires have increased across the whole of Colombia, this fire surge is heterogeneous across regions and by presence or absence of armed groups. Results are generated by region with a focus on the two of the most biodiverse regions for which we also have qualitative insights: Amazon and Andes. Because their national-level command structures are not known with certainty, we consider the presence or absence of one or both of the FARC-EP dissidents and the Gulf Clan neo-paramilitaries at the regional scale. Note that due to the possibility of unobserved confounders, the following results should be interpreted as showing the extent to which the presence or absence of armed groups is correlated with forest fires.

**Fig. 6:**
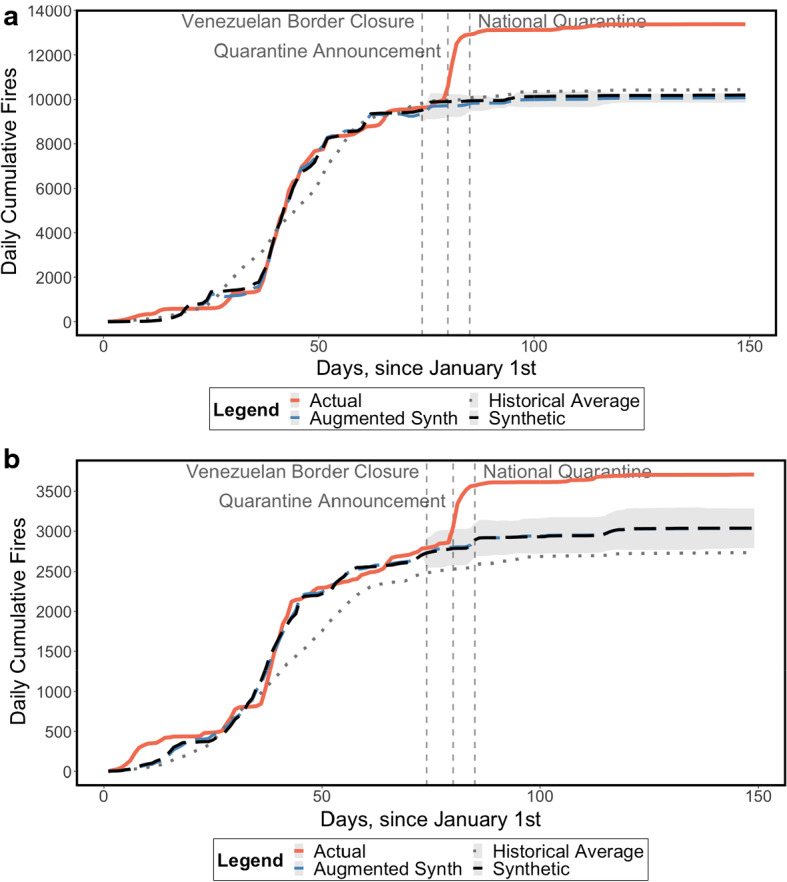
Results for the Amazon region, conditional on armed group presence. **a** Areas with FARC-EP dissidents. *Note*: 95% Confidence Interval around the ASCM series shaded in grey. **b** Areas with no known presence of FARC-EP dissidents or Gulf Clan neo-paramilitaries. *Note*: 95% Confidence Interval around the ASCM series shaded in grey

The Amazon region, which is not naturally fire-prone, experienced an upsurge in forest fires during the process of lockdown (Fig. 6). We observe militarised municipalities controlled by FARC-EP dissidents and municipalities where no known presence of FARC-EP dissidents or Gulf Clan neo-paramilitaries is recorded in our dataset.^7^ Municipalities controlled by FARC-EP dissidents exhibit significantly higher fire trends during the lockdown with respect to the synthetic (3163 more fires as of 28 May) and augmented synthetic (3078–3493 more fires) controls (Fig. 6a).

The contribution of the first month of lockdown to the fire upsurge in the Amazon is even starker than that for the whole country, accounting for 93.8% and 94.7% (the point estimate) with respect to the SCM and ASCM, respectively. Interestingly, and in contrast to the whole country, the fire upsurge begins to manifest after the government’s announcement of a national quarantine on March 20, and levels off once the quarantine took effect, after 24 March.

A significant upsurge in fires, even if more contained, is also observed in municipalities where neither FARC-EP dissidents nor Gulf Clan neo-paramilitaries are known to be present. From Fig. 6b, the lockdown resulted in 672 and 427–923 more fire hotspots with respect to the SCM and ASCM, respectively, again primarily in the first month of lockdown and especially after the announcement of the national quarantine.

**Fig. 7:**
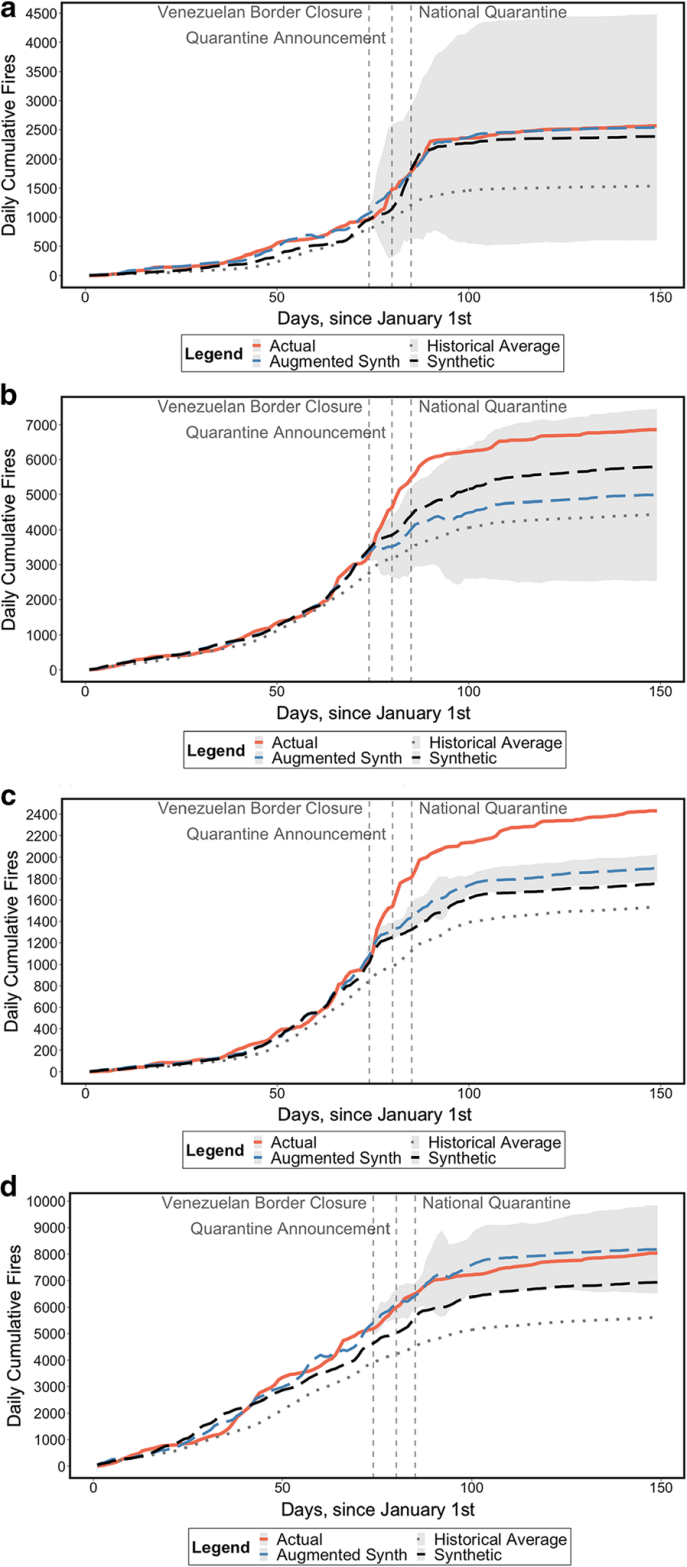
Results for the Andes region, conditional on armed group presence. **a** Areas with FARC-EP dissidents. *Note*: 95% Confidence Interval around the ASCM series shaded in grey. **b** Areas with Gulf Clan neo-paramilitaries. *Note*: 95% Confidence Interval around the ASCM series shaded in grey. **c** Areas with FARC-EP dissidents and Gulf Clan neo-paramilitaries. *Note*: 95% Confidence Interval around the ASCM series shaded in grey. **d** Areas with no known presence of FARC-EP dissidents or Gulf Clan neo-paramilitaries. *Note*: 95% Confidence Interval around the ASCM series shaded in grey

In the Andes (Fig. 7), both FARC-EP dissidents and the Gulf Clan are present in some but not all municipalities, and sometimes together in the same municipalities. Municipalities solely controlled by FARC-EP dissidents do not show significant increases in fires (panel a). Here, the improved performance of the SCM and ASCM with respect to the simple historical mean is apparent and protects us against a false positive result. The ASCM, in particular, shields us against a biased interpretation of the differences in trends between the actual series and the synthetic counterfactuals via the calculation of a 95% confidence interval. As shown in Fig. 7b, the 95% confidence interval does not overlap with the actual trend during the first month of the lockdown, suggestive of a significant fire upsurge in municipalities controlled by the Gulf Clan. By 28 May, however, the observed 2020 rates are comparable with weighted combinations of previous years’ fire seasons.

Andean municipalities that register the compresence of FARC-EP dissidents and the Gulf Clan neo-paramilitaries experienced an upsurge in fires, which is both dramatic and significant (panel c), albeit on a much smaller scale than the increases observed in the Amazon. Here, the null hypothesis of no lockdown effects is rejected in favour of the alternative of an increase accounting for 412–658 more fires with respect to the ASCM as of 28 May.

There are Andean municipalities that have no known presence of either the Gulf Clan or the FARC-EP dissidents, at least according to our dataset (panel d). The ASCM improves significantly upon the SCM and guarantees a good pre-treatment fit. We identify a zero effect from the lockdown in driving up fire rates, with the actual series sitting within the 95% confidence interval around the ridge-augmented synthetic counterfactual.

The other regions (Caribbean, Orinoco, Pacific), the results of which are shown in “Appendix”, display heterogeneity in terms of forest fire trends. In the Caribbean region (Appendix Fig. 13), the presence of either the Gulf Clan alone (panel a), or in combination with FARC-EP dissidents (panel b), is correlated with an upsurge of forest fires during the lockdown, although the counterfactuals are subject to wide uncertainty when we consider the 95% confidence interval around the ASCM fire trends. Interestingly, the patterns observed in the Caribbean are, to some extent, also observed in the Orinoco (Appendix Fig. 14b). The presence of FARC-EP dissidents alone is, similar to the Andes (Fig. 7a), not associated with a fire upsurge (Appendix Fig. 14a). Yet, municipalities where neither FARC-EP dissidents nor the Gulf Clan are known to be present (Appendix Fig. 14d), appears to be associated with a huge fire upsurge (4712 to 6501 fires with respect to the ASCM as of 28 May). The Gulf Clan is omnipresent in the Pacific (Appendix Fig. 15) and similar to the Caribbean and Orinoco regions, this particular armed group is associated with an upsurge of fires during the lockdown.

## Discussion

In this section, we discuss how and why the covid-19 outbreak in Colombia and its lockdown might relate to the particularly large and significant upsurge of fires observed in early-2020. We begin with a number of general, possible mechanisms before exploring those that might help explain spatial heterogeneity in our econometric results with respect to the Amazon and Andean regions, and the role of armed groups.

The covid-19 outbreak is likely to have had the general effect of reducing individuals’ incentives to travel and interact with other people, thus changing behaviour. A mandatory lockdown goes a step further by imposing a potential cost on individuals if moving around and interacting with other people breaks any laws punishable by, e.g. fines. In Colombia, people increasingly became less mobile after 14 March even though the national, mandated lockdown (‘national quarantine’) did not come into effect until 25 March. After 24 March, mobility actually began picking up again.

Further costs are imposed when lockdowns reduce jobs and incomes. If this reduces the ability or willingness to purchase commodities, such as timber and beef, at given prices, then any resultant fall in demand could help alleviate pressure on forests. As of June 2020, the effects of the covid-19 pandemic on commodity markets appear to have been mixed (Mongabay 2020). If lockdowns prevent farmers and other actors from clearing forests, e.g. for new cattle pastures, this may also reduce pressures on forests, at least in the short term.

Our econometric results suggest that the opposite happened in Colombia, indeed that the lockdown may have even increased incentives to start fires illegally. This could occur if there is a fall in the cost of getting caught due to weaker forest law enforcement, especially in forest areas where governance is already fragile. Reports of weakening law enforcement in Latin America’s forests since the covid-19 outbreak began (e.g. BBC 2020; FCDS 2020b; El Pais 2020) suggests the possibility that the behaviour of Colombia’s enforcement agencies might have changed in response to the outbreak and/or lockdown.

We found that Colombia’s lockdown is associated with an upsurge in forest fires between 14 March and 24 March, that is, before the start of the national quarantine yet during the period when people were less mobile. Just after the announcement of the national quarantine on 20 March, both the Chief Prosecutor’s Office and the regional environmental agencies strictly limited or stopped their officials’ field visits, including to deforestation hotspots, not only to protect themselves but also to shield local communities. The armed forces and other agencies involved in Operation Artemisa partially suspended their operations against deforestation, although it is likely that this suspension occurred before the covid-19 outbreak given that the last known, reported operation took place in October 2019. Also, the suspension was apparently motivated by criticism of Operation Artemisa from civil society and the media as well as a new focus on the forced eradication of coca in the Inter-Andean forests (see below).

Along with the possibility that the upsurge of forest fires occurred due to a slowdown of state enforcement, there are a number of alternative explanations. First, forest fires might have been started by farmers and landless people in anticipation of being locked down under the national quarantine. Second, the period after 14 March was already a time when the attention of the media (and government) was focused exclusively on the pandemic. Although plausible, we have little further information on these two explanations and hence, focus our discussion below on a third possible explanation, namely the mobilization of armed groups.

In parts of the Colombian Amazon region, where the state already had a limited presence, armed groups and criminal networks, described by local environmental organisations as ‘land grabbers’ and ‘mafias come bosque’ (forest-devouring mafias) (Krause 2019), took advantage of the lockdown to strengthen their territorial control and expand their activities, particularly forest-based ones (FCDS 2020b). The underlying motivation of the armed groups and criminal networks was to profit from deforestation and generate income, e.g. from cattle ranching. Yet, our econometric analysis suggests that the upsurge in fires in the Amazon, which occurred at around the same time as the slowdown in state enforcement activities, was similar regardless of whether or not municipalities were under the control of FARC-EP dissidents. But as we lack data showing the location of municipalities controlled by criminal networks, we cannot evaluate whether, in municipalities where FARC-EP dissidents were absent, the fire upsurge might be correlated with any of these criminal networks.

That FARC-EP dissidents have strengthened their territorial control and influence in the absence of effective government control in the Amazon region, have expanded to new areas of the Amazon and have taken advantage of the lockdown to ‘burn the jungle’, is increasingly well-documented (Semana Sostenible 2020). The National Natural Parks System temporarily closed all its parks on 16 March 2020 but in February, before the lockdown, FARC-EP dissidents forced the government to remove its rangers from a number of parks in the Amazon (FCDS 2020b). Also, reports suggest that, pre-lockdown, FARC-EP dissidents collaborated with other actors to occupy and clear forest in La Macarena National Park, and local people in forest reserve areas were coerced by FARC-EP to cut or burn down large forest areas for the expansion of cattle ranching and coca production. Such activities are known to have continued during the lockdown.

The tightening of control by armed non-state actors in the Colombian Amazon has made it more difficult for civilian government agencies and NGOs to operate in the region. For the first time, FARC-EP dissidents have threatened the Amazon Vision programme—the Colombian government´s programme for sustainable development in the Amazon—as well as a number of other developmental and environmental actors, including NGOs and agencies affiliated with international development schemes.

Our econometric results for the Andes are heterogeneous. In some areas, the forests were unaffected, either by logging or fire, particularly in municipalities where neither the Gulf Clan nor the FARC-EP dissidents had control, for example, in the highland Andean forests of Cundinamarca. In this part of the high Andes, the local authorities closed the inter-municipal roads during the lockdown thus interrupting the transport of cargo products, except for food, fuels and medicines. Affected commodities included timber, mined extractives such as sand, limestone, and coal, and other resources pertaining to the construction sector, which often involve dredging or grinding of the Andean mountains.

Municipalities in the Andes controlled by the Gulf Clan, particularly those controlled by both the Clan neo-paramilitaries and FARC-EP dissidents, experienced a sharp and significant increase in fires during the lockdown. Whether these two groups were in conflict or working with one another is unclear. Beyond these two groups, there are also other armed groups implicated in the starting of fires. For example, the municipality of Puerto Boyacá (see Appendix Fig. 16) has a long history of being militarised^8^ and was partially abandoned by the government after 2016. Since 2016, organized criminal networks, along with the Gulf Clan neo-paramilitaries, have been in dispute over control of Puerto Boyacá. An important transit zone for drug trafficking in the lowland Andean forests, the national army entered Puerto Boyacá in early-April 2020 to carry out operations to eradicate coca crops and dismantle cocaine laboratories.^9^ Forced eradication involved burning part of the surrounding forest, a process that has occurred in other coca growing areas in the Inter-Andean forests.

## Conclusion

In the context of what is a rapidly developing situation in Colombia, our study is an early contribution regarding whether and how a common policy response to the covid-19 outbreak, namely the lockdown, influences forest fire rates. Our econometric analysis clearly exposed the abnormality of the March–May 2020 fire season. Indeed, the maximum spike in fire observations in Colombia is usually observed around January–February, a trend from which 2020 does not diverge. Yet, as our results show, the 2020 fire season exceeds the intensity of the fire seasons in the previous 8 years by a significant and alarming amount. Although fires occur naturally in some of Colombia’s ecosystems, such as those in the Caribbean and Orinoco regions, this upsurge generated additional carbon dioxide emissions and could be potentially detrimental to biodiversity. Moreover, damaged and degraded forests are associated with an increased risk of future virus spillovers (e.g. Rulli et al. 2017; Olivero et al. 2017).

The public policy implications of the fire upsurge will become clearer as time passes. That said, it is already clear that an increase in carbon dioxide emissions may make it harder for Colombia to meet its ambitions to reduce emissions from deforestation and forest degradation (REDD+). Other policy implications will emerge with further research. First, future work should be able to confirm whether our results hold with the inclusion of climatic data, as and when such data become available. Alternative explanations for the upsurge could also be evaluated, including the fact that the upsurge occurred while the country was preoccupied with the pandemic and the possibility that forest fires were started in anticipation of being locked down. The question then is who might have started these fires. We conjecture that it is likely to have been the same actors who were implicated in activities that involved the burning of forests prior to the covid-19 outbreak. This includes poorer farmers and the landless—whether they had agency or not—but also larger landowners, criminal networks and armed groups.

From our regional analysis, territories controlled by the Gulf Clan neo-paramilitaries, either alone or with FARC-EP dissidents, experienced a significant increase in fire rates during the lockdown. This analysis could be improved with detailed data on the Clan, along with other armed groups, criminal networks, assorted mafias, and their activities. The involvement of some of these groups in the production of high-value commodities, like beef and cocaine, is likely to be central to incentivising forest clearing behaviour. Such incentives could be boosted by reduced government capacity to monitor and enforce the rules against illegal fire-setting. The Colombian government prior to the lockdown not only monitored fire hotspots and deforestation but also the armed groups themselves. Thus, if lockdown-induced reductions in the government’s monitoring and enforcement capacity has played a role in increasing the incentives of armed groups to clear forest, it is imperative that such capacity is reinstated when the covid-19 outbreak recedes.

## Figures and Tables

**Fig. 8:**
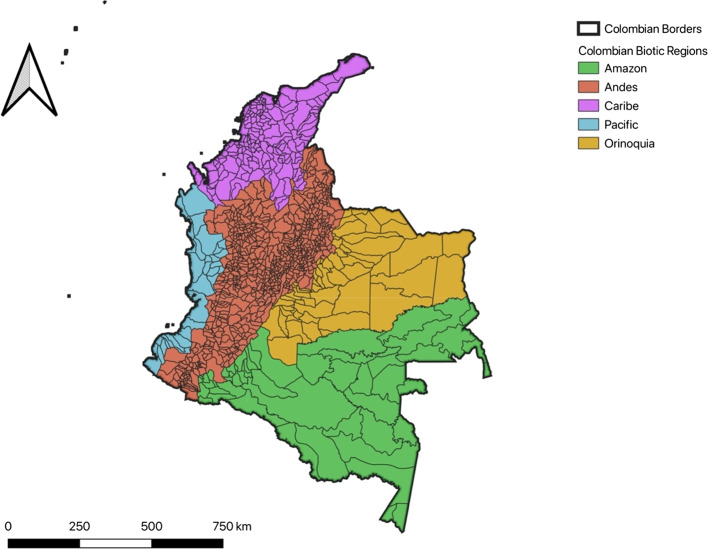
Colombia’s biotic regions. *Note*: areas within regions denote the boundaries of municipalities

**Fig. 9:**
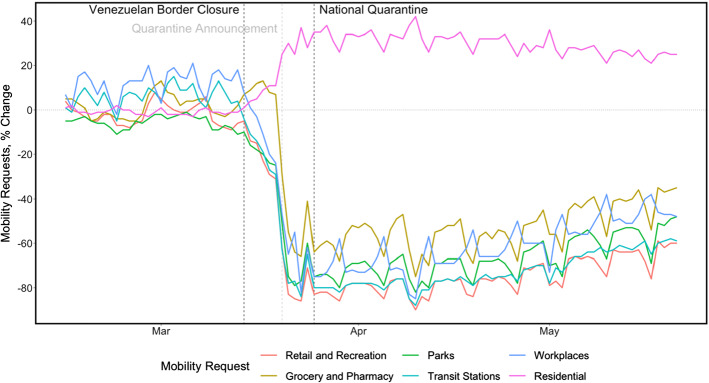
Mobility by destination in Colombia, 2020. *Source*: Google Mobility Reports (https://www.google.com/covid19/mobility/)

**Fig. 10:**
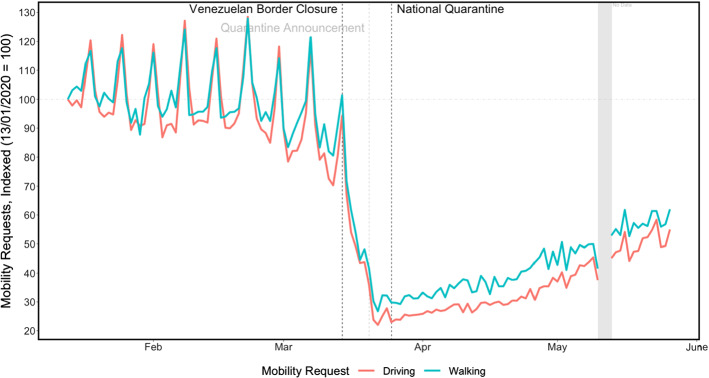
Mobility, walking or driving, Colombia 2020. *Source*: Apple Mobility Reports (https://www.apple.com/covid19/mobility)

**Fig. 11:**
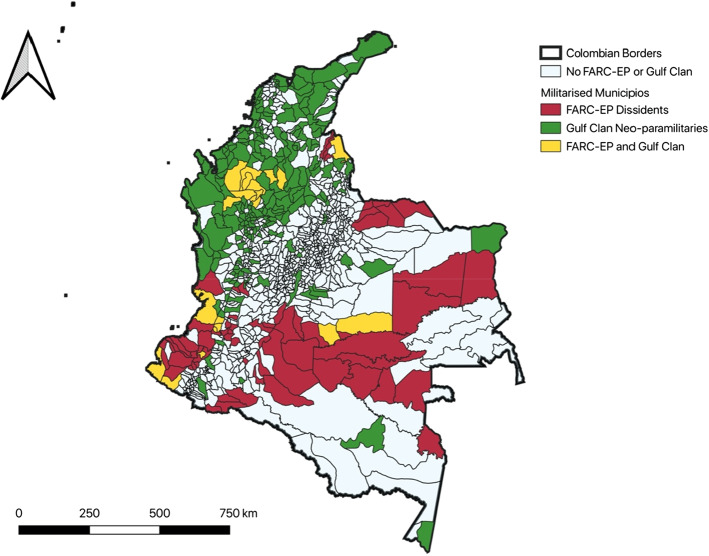
Known locations of FARC-EP dissidents and the Gulf Clan in Colombia by municipality, 2019. *Source*: Peace and Reconciliation Foundation (PARES), and Authors’ elaboration

**Fig. 12:**
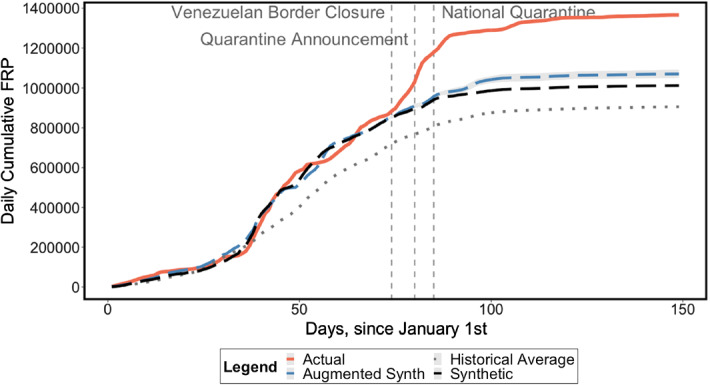
Alternative dependent variable, Fire Radiative Power (FRP). *Note*: 95% Confidence Interval around the ASCM series shaded in grey

**Fig. 13:**
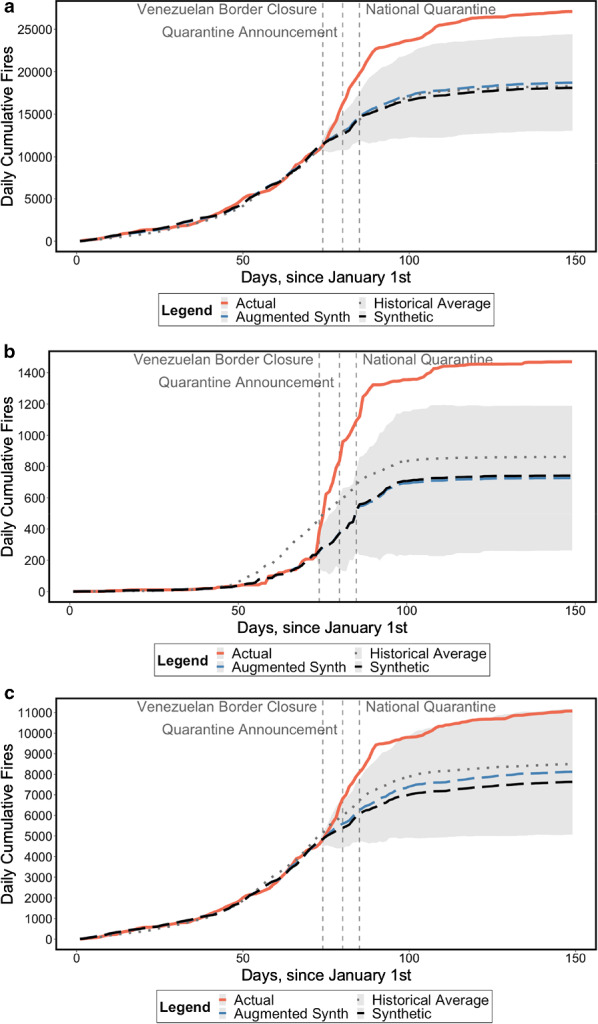
Results for the Caribbean region, conditional on armed group presence. **a** Areas with Gulf Clan neo-paramilitaries. *Note*: 95% Confidence Interval around the ASCM series shaded in grey. **b** Areas with FARC-EP dissidents and Gulf Clan neo-paramilitaries. *Note:* 95% Confidence Interval around the ASCM series shaded in grey. **c** Areas with no presence of FARC-EP dissidents or Gulf Clan neo-paramilitaries. *Note*: 95% Confidence Interval around the ASCM series shaded in grey

**Fig. 14:**
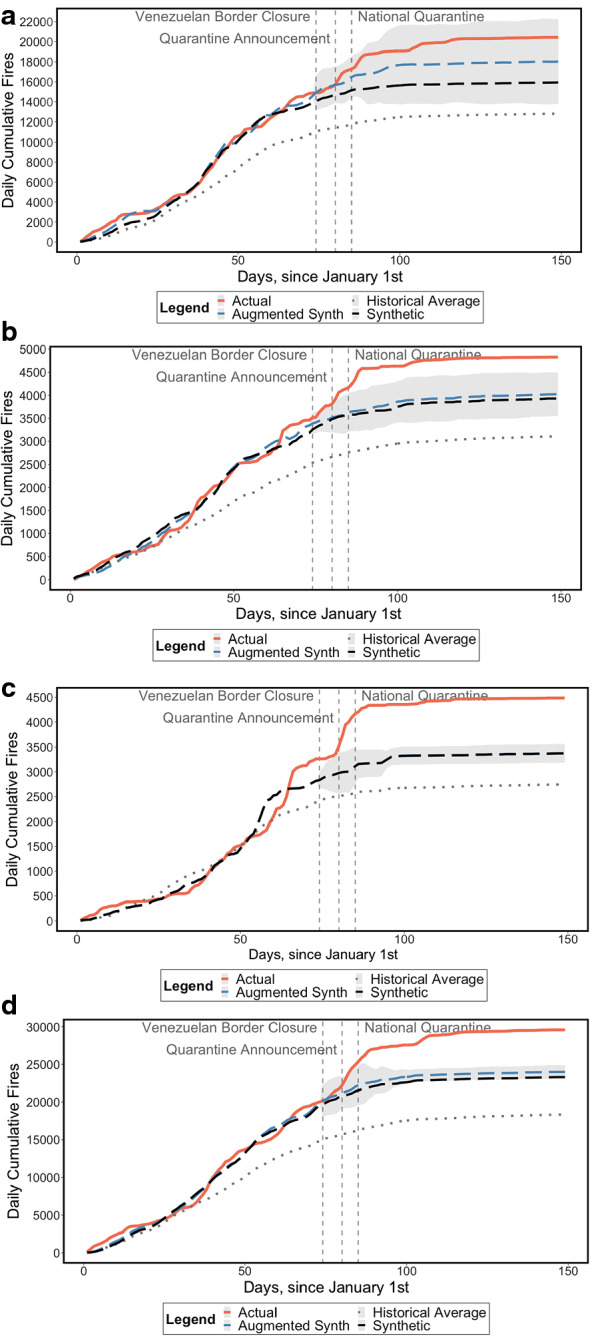
Results for the Orinoco region, conditional on armed group presence. **a** Areas with FARC-EP dissidents. *Note*: 95% Confidence Interval around the ASCM series shaded in grey. **b** Areas with Gulf Clan neo-paramilitaries. *Note*: 95% Confidence Interval around the ASCM series shaded in grey. **c** Areas with FARC-EP dissidents and Gulf Clan neo-paramilitaries. *Note*: 95% Confidence Interval around the ASCM series shaded in grey. No matching was possible, either with SCM or ASCM and hence, we cannot claim parallel trends. The results are not valid and an effect cannot be identified. **d** Areas with no presence of FARC-EP dissidents or Gulf Clan neo-paramilitaries. *Note*: 95% Confidence Interval around the ASCM series shaded in grey

**Fig. 15:**
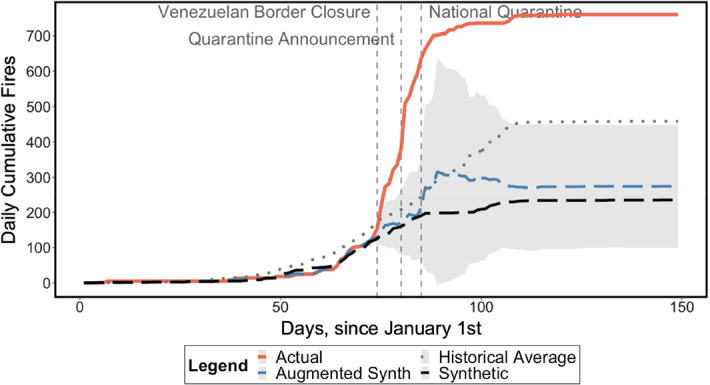
Results for the Pacific region, conditional on armed group presence. **a** Areas with Gulf Clan neo-paramilitaries. *Note*: 95% Confidence Interval around the ASCM series shaded in grey

**Fig. 16:**
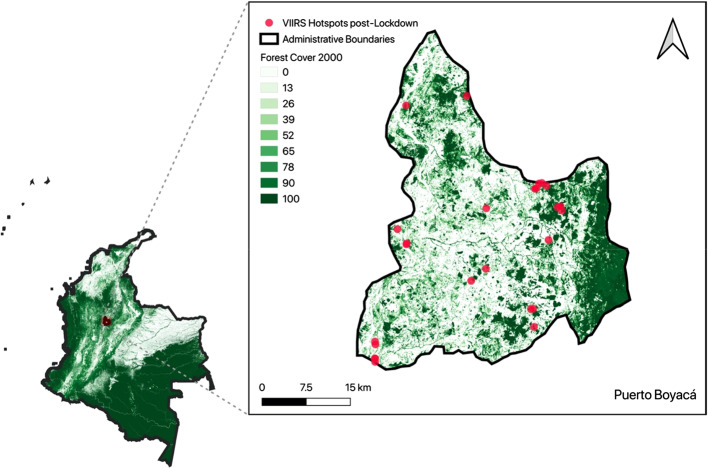
Location of fire hotspots in Puerto Boyacá, 14 March–28 May 2020. *Source*: Hansen et al. (2015), NASA Goddard Space Flight Center, Ocean Ecology Laboratory, Ocean Biology Processing Group, 2020. Visible and Infrared Imager/Radiometer Suite (VIIRS)

## Appendix

See Figs. 8, 9, 10, 11, 12, 13, 14, 15 and 16.

## Footnotes

1 The ‘old’ paramilitary groups were demobilized in 2004–2006 but some of the members of these groups joined forces with paramilitaries that chose not to demobilize to create smaller and more adaptable military organizations, known as neo-paramilitaries, BACRIM or Armed Organized Groups (GAOs).

2 There are different types of protected area in Colombia: National Natural Parks, Regional Natural Parks, National Forest Reserves, Integrated Management Districts, Soil Protection Districts, Natural Reserves and Indigenous Territories.

3 Available from: https://modis.gsfc.nasa.gov/data/dataprod/ and https://earthdata.nasa.gov/earth-observation-data/near-real-time/download-nrt-data/viirs-nrt.

4 Link: http://sites.exeter.ac.uk/bioresilience/.

5 This is reassuring because these three fire seasons have the largest numbers of fires in our dataset. Hence, 2018, 2013 and 2016 are best-positioned to reproduce the 2020 season, respectively, receiving weights of 0.508, 0.437 and 0.055. All the other years receive next-to-zero weights.

6 2019 is the fourth-highest fire year in the record thereby validating the performance of the ASCM.

7 Two municipalities are controlled by the Gulf Clan, as can be seen in Appendix Fig. 11, but their cumulative fire count never exceeds 60 between January and June 2020. Therefore, we exclude them from the dataset.

8 Puerto Boyacá hosted a government-sponsored experiment, a ‘laboratory of military operations’ during the 1980s, in an effort to deprive the FARC-EP of territorial control by using paramilitary groups. This model was exported to other parts of the county, and is known as the Puerto Boyacá paramilitary model. Throughout the 1990s, this model was expanded to other Colombian regions. New paramilitary blocs were established, which incriminated civilians, accusing them of being guerrilla collaborators, perpetuating massacres on this basis, and exercising control over the local economy.

9 This occurred without consultation with, or participation of, local people or their organizations. Part of the 2016 peace agreement between the Colombian government and FARC-EP includes a right granted to local people to participate in voluntary programmes to eradicate illicit crops. However, during the lockdown the government preferred to focus its efforts on the forced eradication of illicit crops without the involvement of local people.
